# Identification of Immunodominant Proteins of the *Leishmania (Viannia) naiffi* SubProteome as Pan-Specific Vaccine Targets against Leishmaniasis

**DOI:** 10.3390/vaccines11071129

**Published:** 2023-06-21

**Authors:** Prisciliana Jesus-Oliveira, Luzinei Silva-Couto, Nathalia Pinho, André Teixeira Da Silva-Ferreira, Leonardo Saboia-Vahia, Patricia Cuervo, Alda Maria Da-Cruz, Adriano Gomes-Silva, Eduardo Fonseca Pinto

**Affiliations:** 1 Laboratório Interdisciplinar de Pesquisas Médicas, Instituto Oswaldo Cruz, Fundação Oswaldo Cruz, Rio de Janeiro 21040-360, Brazil; 2 Laboratório de Pesquisa em Leishmanioses, Instituto Oswaldo Cruz, Fundação Oswaldo Cruz, Rio de Janeiro 21040-360, Brazil; 3 Rede de Pesquisas de Neuroinflamação do Rio de Janeiro, Instituto Oswaldo Cruz, Fundação Oswaldo Cruz, Rio de Janeiro 21040-360, Brazil; 4 Laboratorio of Toxinologia, Instituto Oswaldo Cruz, Fundação Oswaldo Cruz, Rio de Janeiro 21040-360, Brazil; 5 Laboratório de Vírus Respiratórios e Sarampo, Laboratório de Referência para COVID-19 (World Health Organization), Instituto Oswaldo Cruz, Fundação Oswaldo Cruz, Rio de Janeiro 21040-360, Brazil; 6 Rede de Pesquisas em Saúde, Fundação de Amparo à Pesquisa do Estado do Rio de Janeiro, Rio de Janeiro 20020-000, Brazil; 7 Disciplina de Parasitologia, Departamento de Microbiologia, Imunologia e Parasitologia, Faculdade de Ciências Médicas, Universidade Estadual do Rio de Janeiro, Rio de Janeiro 20550-170, Brazil; 8 Instituto Nacional de Ciência e Tecnologia em Neuroimunomodulação (INCT-NIM), Rio de Janeiro 21040-900, Brazil; 9 Laboratório de Pesquisa Clínica em Micobacterioses, Instituto Nacional de Infectologia Evandro Chagas, Fundação Oswaldo Cruz, Rio de Janeiro 21040-360, Brazil

**Keywords:** Leishmaniasis, *L. (Viannia.) naiffi*, immunodominance, reverse vaccinology, pan-specific vaccine

## Abstract

*Leishmaniasis* is a wide-spectrum disease caused by parasites from *Leishmania* genus. A well-modulated immune response that is established after the long-lasting clinical cure of leishmaniasis can represent a standard requirement for a vaccine. Previous studies demonstrated that *Leishmania (Viannia) naiffi* causes benign disease and its antigens induce well-modulated immune responses in vitro. In this work we aimed to identify the immunodominant proteins present in the soluble extract of *L. naiffi* (sLnAg) as candidates for composing a pan-specific anti-leishmaniasis vaccine. After immunoblotting using cured patients of cutaneous leishmaniasis sera and proteomics approaches, we identified a group of antigenic proteins from the sLnAg. In silico analyses allowed us to select mildly similar proteins to the host; in addition, we evaluated the binding potential and degree of promiscuity of the protein epitopes to HLA molecules and to B-cell receptors. We selected 24 immunodominant proteins from a sub-proteome with 328 proteins. Homology analysis allowed the identification of 13 proteins with the most orthologues among seven *Leishmania* species. This work demonstrated the potential of these proteins as promising vaccine targets capable of inducing humoral and cellular pan-specific immune responses in humans, which may in the future contribute to the control of leishmaniasis.

## 1. Introduction

Leishmaniasis is a complex of diseases most important in four eco-epidemiological regions of the world: the Americas, East Africa, North Africa, and West and South-East Asia, according to the World Health Organization (WHO) [1]. It is caused by more than 20 species of *Leishmania* parasites, which are transmitted by dozens of species of sandfly phlebotomine insects. Inoculation of an infective form of the *Leishmania* parasite (metacyclic promastigotes) through the skin of the host can result in one of several clinical forms of the disease. Depending on the *Leishmania* species and host susceptibility factors, infection can lead to cutaneous (localized, mucosal, or diffuse) or visceral leishmaniasis. It is estimated that there are 50,000 to 90,000 new cases of visceral leishmaniasis (VL) annually. It is a potentially fatal disease. Cutaneous leishmaniasis (CL) affects approximately 600,000 to 1 million people worldwide [2]. These facts make the development of vaccines against leishmaniasis a priority.

Although no vaccine against leishmaniasis has been approved for use in humans, different classes of anti-leishmaniasis vaccines have been developed by many research groups around the world [3]. Among the research strategies, subunit vaccines have emerged as an alternative for the control of leishmaniasis. They have several advantages, including more predictable safety than other vaccine platforms, because they do not contain replicative components, and they are amenable to molecular characterization and rational design, as well as modification or deletion of cytotoxic and poorly immunogenic subdomains. Industrial production of the second generation is scalable, and antigens can be designed and formulated for improved thermostability, reducing the cost of delivery to increase vaccine affordability and access [4]. After decades of research, more than 30 protein subunits of *Leishmania* have been isolated, characterized, and tested as polyepitope vaccine candidates [5].

Most of these designed vaccines are directed against one of the clinical forms of leishmaniasis or against infection by one species of parasite. Nevertheless, most of the endemic areas for leishmaniasis have co-occurrence of at least two species of *Leishmania*, with up to eight species detected in sympatry, presenting a challenge for disease control [6]. As of 2021, both CL and VL are endemic to 71 countries, according to WHO [1]. In view of this, the pan-specific approach has recently been considered by several anti-leishmaniasis vaccine research groups. Cross-protection is one of the phenomena underpinning this choice [7]. A pan-specific vaccine type may be able to reproduce the pre-exposure cross-protection conditions of populations living in endemic areas of sympatric species causing CL and VL diseases, such as leishmaniasis [8].

Previously we showed that the total *L. (L.) amazonensis* extract (LaAg) administered through different mucosal routes induced protection against *L. (L.) major* infection in a murine model and against *L. (V.) braziliensis* infection in a hamster model [9,10]. Romano et al. (2015) [11] demonstrated heterologous protection by leishmanization with *L. major* against visceral leishmaniasis caused by *Leishmania (Leishmania) infantum* in a mouse model. Second-generation vaccines have also shown promising results in terms of pan-specific protection. Goto et al. (2011) [12] demonstrated protection by a recombinant polyprotein in murine models of VL (*L. infantum*) and CL (*L. major*). Studies with centrin-deficient *L. mexicana* showed protection against *L. donovani* infection in a hamster model [13].

Computational prediction has been the main tool for the mapping of immunodominant proteins or peptides, allowing the identification of high-affinity links between *Leishmania* peptides and host molecules, such as human leukocyte antigen (HLA) class I (in the case of epitopes presented to TCD8+ lymphocytes), HLA class II (for epitopes presented to TCD4+ lymphocytes), and B-lymphocyte antigen receptors [14]. Epitopes must be promiscuous and bind to various HLA molecules that represent the ethnic diversity of populations in endemic areas of the world. Thus, these epitopes are considered better vaccine targets [15,16].

Several studies strongly show that immune protection against leishmaniasis is associated with a well-modulated and regulated response [17,18,19] and thus represents a standard requirement for an anti-leishmaniasis vaccine. Which *Leishmania* antigens could induce this immune-protective and long-lasting pattern of response?

Our hypothesis is that *L. naiffi* antigens can be used to compose a pan-specific vaccine formulation for the control of leishmaniasis based on the following characteristics: (i) this species causes a benign disease, with no reports of clinical forms associated with hyperactivation of the immune response (mucosal form) or anergy (diffuse cutaneous form); (ii) its antigens are capable of inducing well-modulated immune responses in vitro [20]; and (iii) *L. naiffi* proteins share peptides capable of being cross-recognized by sera from patients infected with different species of the parasite [20,21].

To test this hypothesis, we constructed the entire design of this study by establishing protein-mining criteria. Antigenicity, abundance, low similarity to human host proteins, immunodominance, and conservation across *Leishmania* species were chosen as the baseline to address some of the key requirements of a pan-specific anti-leishmanial vaccine target, as mentioned above. We identified the *L. naiffi* soluble antigenic fraction by immunoblotting with the cured sera of CL patients. The proteins from this fraction were identified using a mass spectrometry-based approach, and by in silico methodologies, we evaluated the binding potential and degree of promiscuity of immunodominant epitopes for HLA molecules and B-cell receptors (BCRs) to finally propose a group of proteins that could be used to create a vaccine against different *Leishmania* species.

## 2. Materials and Methods

### 2.1. Ethics Statements

The patient sera samples were used under approval by the Ethics Committee of the Oswaldo Cruz Foundation (protocols 291/05 and 206/03). In accordance with the Brazilian Law of Biodiversity, this study was registered at SisGen A9FA29D.

### 2.2. Parasite Culture and Isolation of Soluble Extract (sLnAg)

The promastigote form of a strain of *L. naiffi* (MDAS/BR/1979/M5533) was cultured to the stationary phase at 26 °C in Schneider’s Insect Medium (Sigma-Aldrich, St. Louis, MO, USA) supplemented with 10% fetal bovine serum (SFB-Cultilab, São Paulo, Brazil) heat inactivated at 56 °C for 50 min at 25 °C, L-glutamine (1 mM/mL), penicillin (200 U/mL), and streptomycin (200 μg/mL). Samples were collected in the stationary phase by centrifugation at 1800× *g* for 5 min.

Total antigen was obtained adapting the method described by Pinto et al. (2003) [10]. Briefly, wild-type *L. naiffi* promastigotes were washed three times by centrifugation, and the pellet was resuspended at a concentration of 2 × 10^8^ parasites/mL in phosphate-buffered saline (PBS) and subjected to fifteen cycles of freezing and thawing. The soluble *L. naiffi* fraction (sLnAg) was obtained from total extract by ultracentrifugation at 100,000× *g* for 30 min at 4 °C in a Beckman Coulter ultracentrifuge (Brea, CA, USA). A protease inhibitor (Roche) was added to this preparation according to the manufacturer’s guidelines. The total protein concentration was determined using a MicroBCA Protein Assay Kit (Thermo Scientific, Waltham, MA, USA) [22]. This procedure was repeated in three independent experiments.

### 2.3. Human Serum

A total of sixteen human serum specimens were used in the present study. Among these samples, fourteen are from volunteer donors from an endemic area (Rio de Janeiro, Brazil) infected with *L. braziliensis* and affected with the cutaneous form who progressed to a stable and long-term recovery. All leishmaniasis patients had their infection confirmed clinically, epidemiologically, immunologically, and parasitologically. Of all used samples, four were from patients who progressed to spontaneous remission and ten from those cured following treatment. The treatment proceeded according to the guidelines of the Brazilian Ministry of Health (15–20 mg/kg/day of Sb^+5^ for 20–30 days). All these patients had been clinically cured for at least 5 years at the time of serum collection and were followed up for this period at Instituto de Pesquisa Clínica Evandro Chagas (IPEC, FIOCRUZ). As positive controls, sera from 2 patients with active cutaneous leishmaniasis were used.

### 2.4. Immunoblotting of the sLnAg Extract

To access the molecular weight range with the highest frequency of antigens recognition by antibodies, sLnAg (20 µg/well) was fractionated by 10% sodium dodecyl sulfate-polyacrylamide gel electrophoresis (SDS–PAGE). Polypeptides were then transferred to a nitrocellulose membrane at 240 mA for 20 min and submitted to immunoblotting [23]. Membranes were blocked with 0.5% Tween 20 in PBS containing 10% non-fat milk. Human serum samples from CL cured patients and serum from active cutaneous leishmaniasis patients (positive control) were diluted 1:50 and incubated individually with each piece of the membrane. After washing, the pieces were incubated with peroxidase-conjugated human IgG (Sigma) diluted 1:1000. The frequency of antigen recognition by the sera was defined by densitometric analysis of the immunoblotting with the program 2.0.

Furthermore, sLnAg were fractionated under the same electrophoretic conditions described above; proteins were stained with Coomassie Brilliant Blue (Bio-Rad, Hercules, CA, USA [24]) and the bands corresponding to the molecular masses recognized by the sera were manually excised from the gel. This procedure was performed in triplicate.

### 2.5. In-Gel Tryptic Digestion

The proteins were enzymatically digested following procedures previously described [25,26]. Briefly, gel slices were distained three times with 50% acetonitrile and 25 mM ammonium bicarbonate solution (pH 8.0) until all Coomassie Blue and SDS were removed. Proteins were then reduced in 65 mM dithiothreitol (DTT) for 30 min at 56 °C and alkylated with 200 mM iodoacetamide at 25 °C in darkness for 30 min. After washing with 100 mM ammonium bicarbonate, gel slices were dehydrated with acetonitrile and dried in a vacuum centrifuge for 15 min at room temperature. The slices were rehydrated with a solution of 20 ng/µL of sequencing grade modified porcine trypsin (Promega, Madison, WI, USA) in 50 mM NH_4_HCO_3_ and incubated overnight at 37 °C. The peptide solution resulting from the digestion was separated and peptides remaining in the gel were extracted using 1% formic acid in 50% *v*/*v* acetonitrile. Peptides were then desalted and concentrated using Octadecyl C18 membranes (Empore™, 47 mm extraction discs, Thermo Fisher, Waltham, MA, USA), arranged in tips, as previously described [27].

### 2.6. Mass Spectrometry Analysis

Tryptic peptides were analyzed by reversed phase nanochromatography coupled with high-resolution nanoelectrospray mass spectrometry. Initially, for each sample, 4 µL of desalinated peptides was loaded onto a 2 cm long trap column (internal diameter 100 µm) packed with 5 µm 200 A Magic C18 AQ matrix (Michrom Bioresources, Auburn, CA, USA). This was followed by separation in a 10 cm long (75 µm internal diameter) separation column, which was packed with Reprosil-Pur 120 C18_AQ 1.9 µm, and the eluate was delivered directly into a 15 µm empty tip column (made in-house) using laser extractors. Chromatography was performed on an EASY-nLC II instrument (Thermo Fisher). Samples were loaded onto the trap column in 2000 nL/min, and chromatographic separation took place at 200 nL/min. Mobile phase A consisted of 0.1% (*v*/*v*) formic acid in water. Mobile phase B consisted of 0.1% (*v*/*v*) formic acid in acetonitrile. The gradient was as follows: 2 to 40% B over 32 min and 40 to 80% B over 4 min. After maintaining this concentration for another 2 min, the column was re-equilibrated. The eluted peptides were injected directly into the LTQ XL/Orbi/Trap MS spectrometer (Thermo Fisher). The subsequent analysis was performed with the following conditions adjusted: voltage source: 1.9 kV, capillary temperature: 200 °C, and tube lens voltage: 100 V. The full ion trap value was 30,000, and the MSn AGC target value was 10,000. The total AGC target value of the Fourier ion cyclotron transformer (FTMS) was set to 500,000. MS1 spectra were acquired on the Orbitrap analyzer (300 to 1700 *m*/*z*) with a resolution of 60,000 (for *m*/*z* 4,451,200). For each spectrum, the 10 most intense ions were subjected to CID fragmentation (minimum required signal of 10,000; insulation width of 2.5; normalized collision energy of 35.0; activation Q of 0.25; and activation time of 30 s) followed by MS2 acquisition on a linear trap analyzer. The dynamic deletion option was enabled. The parameter settings were as follows: repetition count = 1, repetition duration = 30 s, exclusion list size = 500, duration exclusion = 45 s, and exclusion mass width = 10 ppm.

### 2.7. Data Analysis

For peptide identification, MaxQuant software was used (V.2.0.3.0 [28,29]). The mass spectra were searched against a database containing *L. naiffi, L. panamensis*, *L. braziliensis*, and *L. guyanensis* sequences available in UniProtKB/Swiss-Prot (downloaded in August 2021) plus reversed protein sequences used as decoys, as well as common contaminant sequences [29]. The fragment ion mass tolerance was set to 0.5 Da and the parent ion tolerance to 20 ppm, and the option “matching between runs” was used for searching. Cysteine carbamidomethylation was set as a fixed modification, methionine oxidation and N-terminal acetylation were set as variable modifications, and up to two missed cleavages were accepted. The maximum false peptide and protein discovery rate (FDR) was set to 1%. Data validation and statistical analysis were performed using Perseus software (V. 1.6.1.3) [30]. We applied Student’s *t* test for comparisons between groups. Only proteins identified in at least two of the three biological replicates and with at least two unique peptides per protein were accepted.

**Figure 1:**
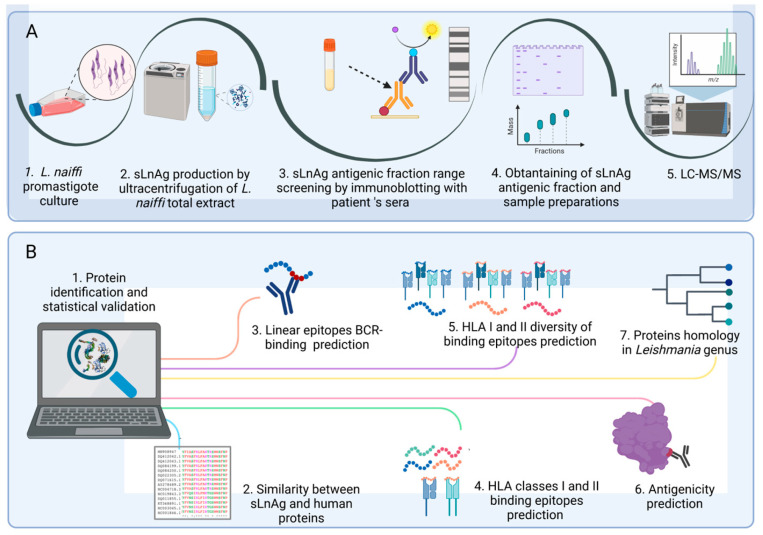
Workflows of the proteomic and bioinformatic analysis. (**A**). Proteomic experiments. Cultured metacyclic promastigotes of *L. naiffi* originated total extract. sLnAg was obtained by ultracentrifugation of the total extract. Groups of sLnAg proteins were separated by SDS-PAGE and incubated with sera from cured CL patients. The molecular weight band with the highest frequency of antigen–antibody complexes was obtained in a new electrophoretic run under the same conditions as the previous one. Following FASP elution, the fractions were subjected to LC-MS/MS. (**B**). Bioinformatics analysis. The proteins of the antigenic fraction of sLnAg were identified from the mass spectra obtained. Subsequently, a protein-mining analysis was performed. We excluded proteins similar to human proteins and selected proteins with more BCR and HLA I and II binding epitopes, as well as greater diversity with respect to HLA allele binding. The selected proteins were analyzed for antigenicity using the Vaxijen algorithm and for conservation in the genus *Leishmania*, criteria to be considered as potential vaccine targets.

A scheme of the proteomic and bioinformatic workflows is provided in Figure 1 (created with BioRender.com). Analyses of antigen fraction detection (immunoblot with patient sera) and prediction of BCR-binding epitopes are related to the humoral response and HLA-binding predicts the antigen presentation to T lymphocytes.

### 2.8. Analysis of Sequence Similarity between Leishmania and Human Proteins

To exclude proteins with a high similarity to human proteins, identified sLnAg protein sequences were searched against human sequences using the Basic Alignment Search Tool (BLAST) (https://blast.ncbi.nlm.nih.gov/Blast.cgi, accessed on 7 March 2022). The similarity classifications of Rost (1999) [31] and Chung et al. (1996) [32] were used. Thus, proteins with no significant similarity or low similarity (similarity values between zero and 30%) were selected for further analysis. The sequences of *Leishmania infantum* A2 protein (A2) [33], *Plasmodium falciparum*—circunsporozoite protein (CSP) [34], and *Mycobacterium tuberculosis* Ag85A protein (Ag85A) [35] were used as controls in this and subsequent analyses. These control proteins are well-characterized as vaccine targets.

### 2.9. Prediction of Linear Epitopes Recognized by B lymphocytes

*Leishmania* protein sequences whose similarity to human sequences was less than 30% were subjected to predictive analysis of high-affinity linear epitopes recognized by B-cell receptors (BCRs) with the following programs: (i) BepiPred, version 2.0 [36] http://tools.iedb.org/bcell/, accessed on 7 March 2022, with the database IEDB [37] was used to identify high-affinity linear epitopes from crystallized structures of antigen–antibody complexes. (ii) ABCPred, which uses the Bcipep linear epitope database (https://webs.iiitd.edu.in/raghava/abcpred/ABC_method.html, accessed on 20 April 2022) [38,39,40], was used to identify high-affinity linear epitopes based on physicochemical characteristics of the sequences. The default parameters of each program were used to select peptides containing nine amino acids.

### 2.10. Prediction of Class II HLA-Binding Epitopes

We predicted the binding of epitopes to HLA class II molecules with the highest frequency in the general population [41]. Based on the IPD-IMGT/HLA database (https://www.ebi.ac.uk/ipd/imgt/hla/alleles/, accessed on 20 April 2022), using the tool Allele Frequency in Worldwide Populations Database (http://www.allelefrequencies.net/, accessed on 20 April 2022) and the IPD-IMGT/HLA Allele Query Tool [42], the considered haplotypes were as follows:

DRB1*01:01; DRB1*03:01; DRB1*04:01; DRB1*04:04; DRB1*04:07; DRB1*07:01; DRB1*08:02; DRB1*11:01; DRB1*12:01; DRB1*13:01; DRB1*13:02; DRB1*15:01; DRB3*01:01; DRB3*02:02; DRB4*01:01; DRB5*01:01; DPA1*01:03-DPB1*02:01; DPA1*01:03-DPB1*04:01; DPA1*02:01-DPB1*01:01; DPA1*03:01-DPB1*04:02; DQA1*01:01-DQB1*05:01; DQA1*01:02-DQB1*06:02; DPA1*02:01-DPB1*14:01; DQA1*03:01-DQB1*03:02; DQA1*04:01-DQB1*04:02; DQA1*05:01-DQB1*02:01; DQA1*05:01-DQB1*03:01.

The Consensus tool available at the Immune Epitope Database and Analysis Resource (IEDB [43]) and NetMHCIIpan-4.0 software (https://services.healthtech.dtu.dk/service.php?NetMHCIIpan-4.0 [44], accessed on 20 April 2022) were used. The fifteen-amino-acid peptides with binding scores calculated according to the standard parameters and thresholds of the programs were classified as strong or weak binders. Only sequences with higher strong binding epitope counts were considered for subsequent analyses.

### 2.11. Prediction of Class I HLA-Binding Epitopes

Prediction of promiscuous epitopes was performed for the following human leukocyte antigen (HLA) class I alleles, selected for this analysis as previously described: HLA-A*01:01; HLA-A*02:01; HLA-A*02:03; HLA-A*02:06; HLA-A*03:01; HLA-A*11:01; HLA-A*24:02; HLA-A*31:01; HLA-A*68:01; HLA-B*07:02; HLA-B*08:01; HLA-B*35:01; HLA-B*40:01; HLA-B*44:02; HLA-B*51:01; HLA-C*03:03; HLA-C*04:01; HLA-C*05:01; HLA-C*06:02; HLA-C*07:01; HLA-C*07:02; HLA-C*12:03; HLA-A*29:02; HLA-B*35:03; HLA-B*38:01; HLA-C*15:02.

The analysis was performed on the NetMHCpan ([45] http://www.iedb.org, accessed on 11 May 2022) and NetCTLpan1.1 ([46] https://services.healthtech.dtu.dk, accessed on 11 May 2022) servers. We considered predictions of theoretical population coverage values, proteasomal cleavage, transport efficiency associated with antigen processing (TAP), and HLA class I binding strength and affinity. In both analyses, the programs used the standard parameters for 9 amino acids’ epitope selection.

The sequences with the highest number of predicted high-affinity and higher-promiscuity binding epitopes for HLA classes II and I molecules and with binding and linear epitopes recognized by BCR predicted simultaneously by the two predictors were selected for subsequent analyses.

### 2.12. Antigenicity Analysis

To corroborate the immunodominant potential data, the sequences selected from the epitope predictions were used for antigenicity prediction using the VaxiJen algorithm [47,48] (http://www.ddg-pharmfac.net/vaxijen, accessed on 13 September 2022). The relationship between the physicochemical characteristics of a sequence and the probability of antigenicity is represented by a score with a default cut-off of 0.5.

### 2.13. Homology Analysis

Sequences that showed value of antigenicity in the previous analysis were included in this analysis. To verify the homology of the proteins in other *Leishmania* species, this analysis was performed with the Order Kinetoplastida as the target taxon using eggNOG genes, version 5.0 [49] and the OrthoDB database, version 10.1 [50]. The total number of orthologues per immunodominant sequence identified by the two programs was used as the prediction result. Sequences with at least eight orthologs predicted sequences relative to a minimum of five *Leishmania* species were selected for further analysis. We predicted orthologous proteins between potentially immunodominant sLnAgs sequences and thirteen species of the genus *Leishmania* (four of which belong to the *L. Viannia* subgenus).

Selected proteins from this analysis were considered as potential vaccine targets. Basic information on these sequences was collected from the public databases UniprotKB [29] and Interpro (available at https://www.ebi.ac.uk/interpro/, accessed on 1 November 2022) [51].

## 3. Results

### 3.1. Antigenic Fraction from the L. naiffi Soluble Antigen Is between 35 and 70 kDa

**Figure 2:**
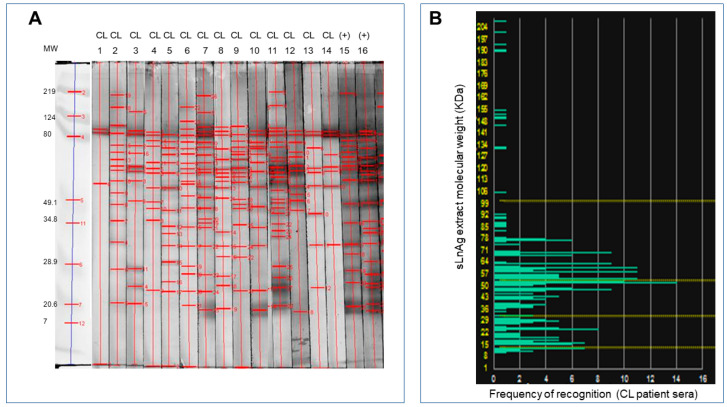
Detection of antigenic proteins in *L. naiffi* by antibody recognition. (**A**). The *L. naiffi* total antigens separated according to molecular weight by electrophoresis were transferred from the agarose gel to nitrocellulose membranes for Western blot analysis. Sera from fourteen volunteer donors from endemic areas for *L. Viannia* with long-term recovery from cutaneous leishmaniasis were incubated with nitrocellulose membranes containing *L. naiffi* antigens (strips 1 to 14) for immunoblotting analysis. Sera from volunteer donors with active disease were used as positive controls (strips 15 and 16). (**B**). Frequency of recognition of antigenic fractions shown on a histogram based on quantitative analysis of the blots. Antigens with a molecular weight of 25 to 78 kDa were recognized most frequently.

To identify sLnAg antigens that could be used to create a pan-specific vaccine formulation, we conducted immunoblotting analysis to evaluate the frequency of antibody response to proteins of these antigens. We observed that antigens with molecular masses ranging from 35 to 70 kDa were commonly recognized by all serum samples from 14 patients with long-term recovery from the *L. braziliensis* endemic area (Figure 2).

Mass spectrometry-based proteomics analysis allowed the identification of a total of 3752 peptides corresponding to 763 protein groups at a 1% FDR (Table S1). Of these protein groups, 393 (51.5%) were identified as having at least one unique peptide and were observed in the three biological replicates; therefore, they were accepted as valid and reliable identifications (Table S2). These proteins were statistically valid for further analysis. Of this set of proteins, 31 (about 8%) are proteins that have not yet been characterized.

### 3.2. sLnAg Proteins with Less than 30% Similarity to Human Proteins Represent 39% of Identified Proteins

The analysis of similarity between the 393 sLnAg proteins and human proteins revealed that only 39% (154/393) of the antigenic sLnAg proteins had less than 30% similarity to human proteins. Therefore, they are in the low-similarity range (Table S3). This means that they are unlikely to have a mimic effect in the human organism. Those proteins were selected for further analysis. These results were similar to those obtained with the already characterized vaccine proteins, considered as controls. The circunsporozoite protein, the Ag85A protein, and the A2 protein showed low similarity to human protein sequences.

### 3.3. sLnAg Proteins Less Similar to Human Proteins Have Predicted BCR-Binding Epitopes

**Figure 3:**
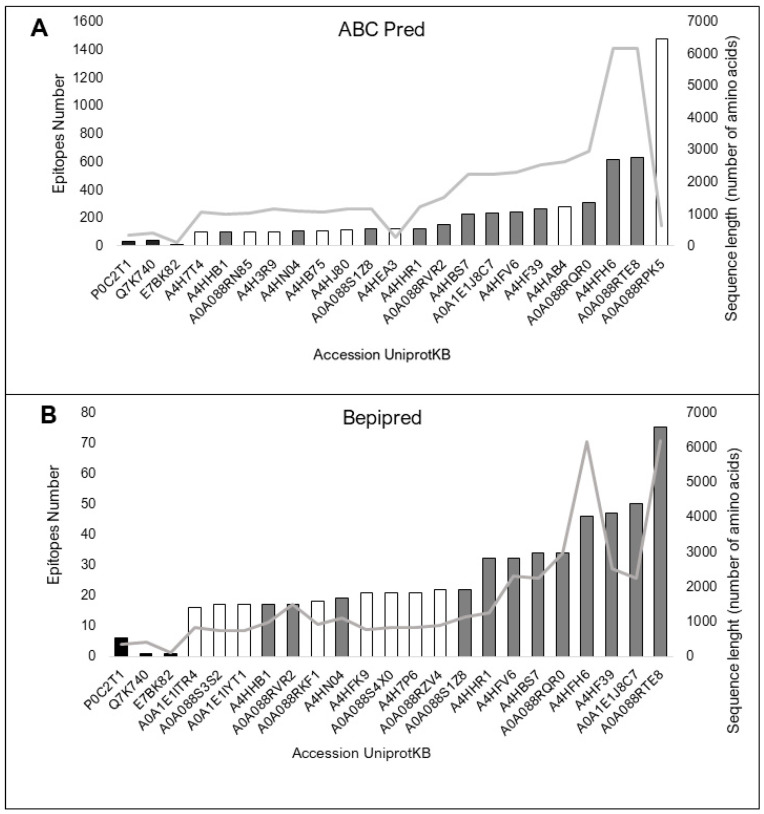
The top twenty proteins in the two BCR-binding epitope prediction methods. The vaccine protein sequences A85A from *M. tuberculosis*, Q7K740 (CSP) protein from *P. falciparum* and E7BK82 (A2 protein from *L infantum*) were used as controls (black bars). Consensus predictions by both programs are shown as grey bars. The sequence size (amino acids) is represented by the light grey line. (**A**). Prediction by the ABCPred web server, based on an artificial neural network algorithm and trained on the Bcipep database. (**B**). Prediction result by the BepiPred web server, based on sequences annotated from antigen–antibody complex crystal structures and from the IEDB, as analyzed by the random forest algorithm.

All 154 proteins showed high-affinity epitopes predicted to be recognized by BCRs by both predictors. However, there were considerable differences in the values between the predictions of the control and sLnAg proteins. This difference can be explained by the divergence in size between the two databases used to predict the BCR-binding epitopes. Bcipep is based on linear epitope sequences (broader), and BepiPred is based on conformational epitopes defined from known crystal structures (narrower). Twenty proteins had more predicted epitopes than the control proteins (Figure 3A,B).

### 3.4. The sLnAg Proteins Have Predicted HLA II-Binding Epitopes and/or HLA I-Binding Epitopes

**Figure 4:**
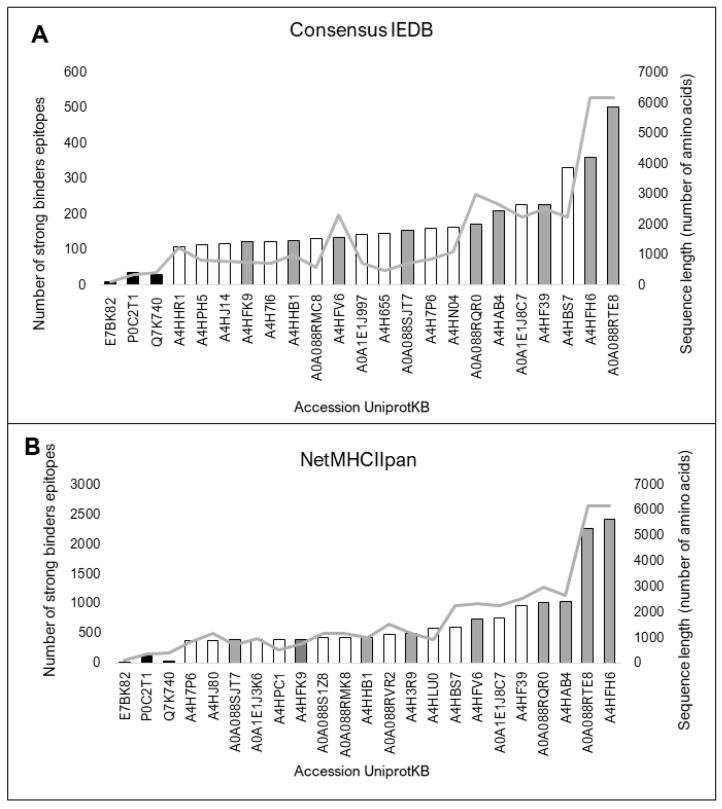
Twenty proteins with the highest predicted number of high-affinity binding epitopes for twenty-seven HLA II haplotypes with high frequency in the global human population. The black bars represent vaccine protein sequences analyzed as control components. Consensus predictions by both programs are shown as grey bars. The sequence size (amino acids) is represented by the light grey line. (**A**) Prediction by Consensus method, a tool that combines high-performance methods available in IEDB, and (**B**) Prediction by NetMHCIIpan, based on an artificial neural network algorithm and trained on the Bcipep database. Both servers use the epitope database IEDB.

The 154 sLnAg sequences with less similarity to human proteins were also analyzed for high-affinity binding epitopes for HLA molecules. All control vaccine protein sequences showed fewer predicted epitopes than the sLnAg sequences by the two programs used. The top 20 sequences in the analyzed set with more predicted epitopes contained more than 100 epitopes per sequence (Figure 4A,B).

**Figure 5:**
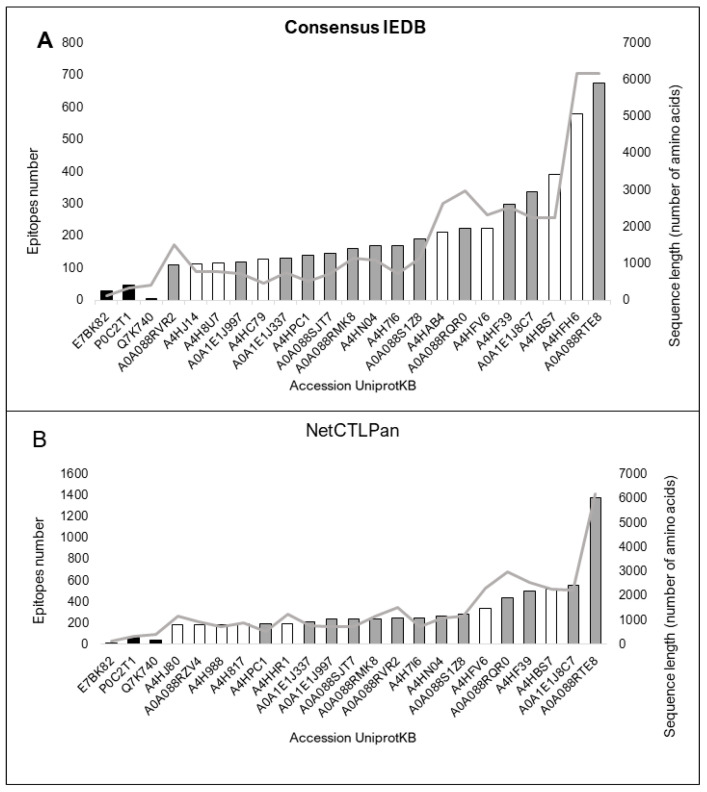
Twenty proteins with the highest predicted number of binding epitopes with high affinity for twenty-six HLA I haplotypes with high frequency in the global human population. The black bars represent vaccine protein sequences analyzed as control components. Consensus predictions by both programs are shown by grey bars. The sequence size (a) (amino acid) represented by the light grey line. (**A**). Prediction by NetCTLpan, based on an artificial neural network algorithm. (**B**). Prediction by NetMHCpan, which combines high-performance predictor data. Both servers were trained on the epitope database IEDB.

**Figure 6:**
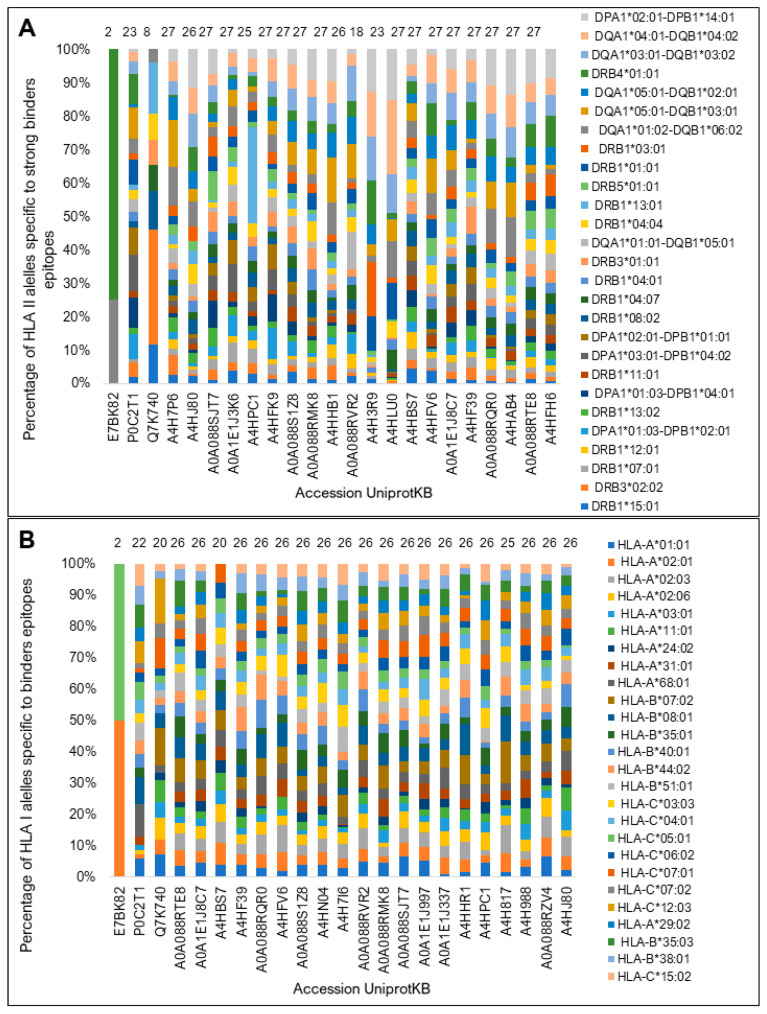
Potential of diversity of binding to HLA diversity (%) for the top twenty and control proteins. (**A**) NetMHCIIpan determined promiscuity percentages of binding epitopes for each HLA-II isotype. (**B**) The NetCTLpan program was used to predict the binding promiscuity to HLA I. The number of HLA II- and I-binding haplotypes predicted for each sequence is shown at the top of the bars. The sizes of the bars represent the percentage of sequences recognizing each isotype corresponding to the color.

We determined that the top 20 sequences contained at least 110 epitopes with a high score for binding affinity for HLA I and exhibited proteasomal processing and transport (Figure 5A,B). The two programs used showed consensus results for 11 of the top 20 sequences. Most of these proteins showed binding potential for all HLA I haplotypes considered in this analysis (Figure 6).

Regarding HLA-binding promiscuity, 14 of the top 20 sequences showed binding potential for all HLA II molecules by the NetMHCIIpan predictor (Figure 6A) and 20 to HLA I molecules according to NetCTLpan (Figure 6B). Only the control sequence Ag85A showed an HLA II- and I-binding promiscuity similar to that of the test sequences. The sequence of the protein A2 showed the lowest diversity binding to both HLA molecules.

### 3.5. Consensus Predictions Determined the Potential Immunodominance of sLnAg Proteins

We selected 24 protein sequences that were predicted by the two programs used in each prediction type, BCR, HLA II, or HLA I. These sequences were considered potentially more immunodominant (Table 1). Of these sequences, seven showed more predicted epitopes in all predictions.

All 24 sequences showed high binding promiscuity for different HLA II and I haplotypes. The highest predicted values were comparable between the control and sLnAg sequences. Despite the large size difference between the control and sLnAg sequences, both generally showed an approximate proportion of predicted epitopes (approximately 1/10) related to the sequence length.

### 3.6. Of the Most Immunodominant Sequences, 20 Are also Antigenic

Interestingly, antigenicity analysis using Vaxijen revealed that 20 of 24 most immunodominant sequences were also antigenic, showing antigenicity values above the 0.5 cut-off value (Table 1). Regarding the protein sequences used as controls, the CSP sequence presented the highest antigenicity score (almost twice the 0.5 cut-off value), whereas the A2 protein showed the lowest one (below the cut-off). The antigenicity score was below cut-off also for seven (7 of 24) of the sLnAg sequences despite being immunodominant (Table 1). Interestingly, every nonantigenic sequence showed more HLA I predicted epitopes.

### 3.7. Potential Vaccine Targets Are the Most Immunodominant and Conserved sLnAg Sequences

**Figure 7:**
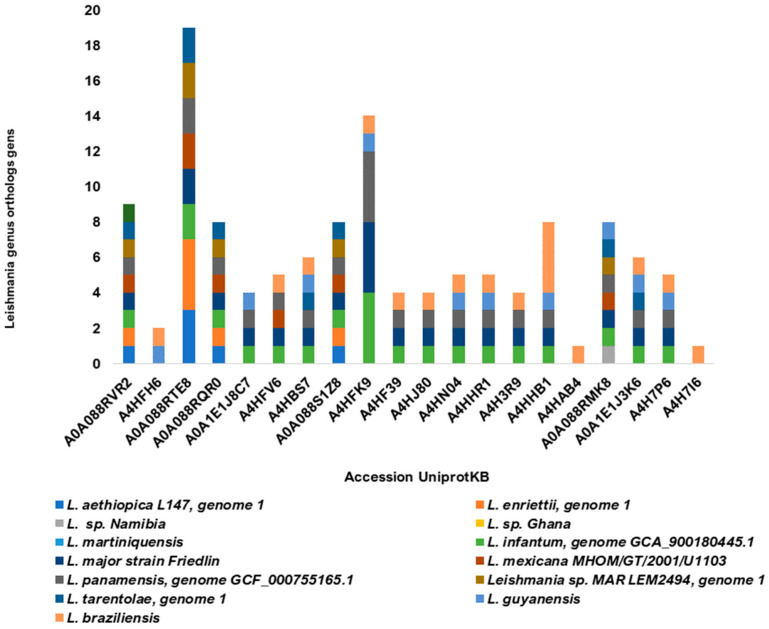
Homology of the twenty most predicted immunodominant sLnAg sequences. The number of proteins homologous to proteins from fourteen *Leishmania* species is represented by the colors of the bars.

The 20 potentially more immunodominant and antigenic sequences analyzed showed orthologous genes in thirteen *Leishmania* species, one of which causes visceral leishmaniasis (*L. infantum*). The selected sequences showed between 8 and 19 homologous genes in at least 5 to 10 *Leishmania* species (Figure 7). Orthologs to both visceral and cutaneous causative species were found in 13 of 20 sequences. Some sequences were found to be too orthologous in species of the genus *Trypanosoma* (data not shown).

This analysis completes the experimental design of computational mining presented in this work. The set of protein sequences selected based on the criteria of abundance, antigenicity, immunodominance, and conservation therefore represent potential pan-specific vaccine targets against leishmaniasis. Groups, families, and other characteristics of the selected proteins are described in Table 2. All selected proteins are found on the databases as predicted sequences. They are also large proteins, and all were identified in species of the subgenus *Viannia: L. panamensis, L. braziliensis,* and *L. guyanensis* [52,53,54].

## 4. Discussion

An ideal vaccine should mimic the immune response pattern that leads to mild disease or disease resolution [55]. Therefore, it is interesting that molecular patterns related to aggressive outcomes are excluded from vaccine compositions. The previous results from our group showed that the infection with *L. naiffi* induces a macrophage activation status that is different from that observed in other *Leishmania* species, leading to slower destruction of the internalized parasite in vitro. In addition, soluble extract of this parasite (sLnAg) very efficiently induces a lymphocyte response in vitro and results in well-modulated IFN-γ production [20]. These findings may be associated with the milder damage to tissues adjacent to lesions than observed in other clinical forms of CL [56,57]. This finding also supported the hypothesis that antigens from *L. naiffi* species may constitute vaccine targets against leishmaniasis. For this reason, in this study, we looked for possible antigens in this species that could be used to make a prototype of a second-generation vaccine.

In this study, the starting mining point was the choice of a soluble antigen for the expression of abundant cytoplasmic and/or secreted proteins, which are related as more antigenic and susceptible to processing and presentation to T cells than membrane proteins [58]. Next, strengthening the already-demonstrated similarity between promastigote and amastigote proteomes [59], we found that sera from CL patients with long-term recovery successfully recognized antigens present in the sLnAg fraction obtained from promastigote cultures.

Mapping of available *Leishmania* proteomes has been the strategy most used by several research groups to identify protein candidates for the development of vaccine targets against leishmaniasis [60,61,62].

The present work focused on antigenicity analysis. In addition, we also considered immunodominance by checking epitope abundance, antigen processing and transport potential, and HLA binding, which together with antigenicity are key conditions for immunogenicity. Further studies in vivo will be needed to address the immunogenicity of these proteins.

Using abundance as one of the selection criteria for target proteins, we analyzed these fractions by proteomics. It is accepted that vaccine protection level results from a combination of several factors including protein abundance, induction of the antigen–receptor immune response, along with antigenicity, immunogenicity, and protein repertoire presented [63,64]. The correlation between higher protein abundance and expression of peptides with higher affinity for HLA molecules has been demonstrated in cancer cells [65]. Additionally, abundance is related to antigen availability for protease-dependent processing in antigen-presenting cells [65]. Thus, among the set of proteins selected in this study, 2 have already been described as among the 20 most abundant protein groups in other subgenus *Viannia* species [27]: the putative calpain-like cysteine peptidase protein and contig, a possible fusion of chromosomes 20 and 34.

Next, to analyze all the abundant proteins we found, we performed a reverse vaccinology methodological design using bioinformatics tools. For this, we chose mining criteria based on the relation between protection against a pathogen and host factors, such as pre-existing immunity, genetic diversity, and antigen cross-recognition, as well as parasite factors including the presence of abundant and immunodominant antigens and their interspecies conservation.

One of the first criteria for mining sLnAg sequences was similarity to human proteins. It is a quantitative feature defined by the percentage of identical amino acids plus those with the same biochemical properties predicted (for example, polarity and functional groups) between two or more compared sequences [31]. This feature is important in developing subunit vaccines to eliminate highly similar host protein sequences that induce a non-specific response through molecular mimicry, cross-recognition of self-proteins by the human immune system, and autoantibody formation [66,67].

Furthermore, we screened for sequences with the highest number of B-cell-binding epitopes. Interestingly, this prediction agrees with the immunoblotting results in vitro showing that potential BCR-binding epitopes in the protein fraction were recognized by antibodies from CL patients with long-term recovery. Several of these sequences showed high numbers of predicted responses. It is already known that TCD4+ lymphocytes play a major role in the adaptive immune response against CL [68]. These cells recognize the intravesicularly processed peptide antigen bound to the HLA II molecule on the surface of APCs.

The tendency or preference for an epitope to be selected for presentation via HLA I or II molecules during the generation of an anti-pathogen immune response constitutes immunodominance. This feature is influenced by epitope–HLA affinity, antigen abundance, promiscuity of antigen binding, and peptide flanking regions [69]. Thus, immunodominance was the next sequence-mining criterion in this study. The number of predicted HLA- and BCR-binding epitopes in a protein is a determinant of this characteristic [14].

The set of most abundant proteins with low similarity to human proteins was subjected to immunoinformatic predictions based on the cellular and humoral adaptative vaccine responses desirable against leishmaniasis. We analyzed the processing of potential antigens. This criterion indicates whether the antigen is correctly cleaved, transported, and exposed on the surface of antigen-presenting cells [70]. Predictors used here revealed that almost twice as many epitopes of sLnAg bind to HLA II molecules than to HLA I molecules, which is expected since the CD4+ T cell response is predominant in leishmaniasis because of intravesicular infection. In this set of predicted HLA II-binding epitopes, most bind HLA II haplotypes found in endemic areas of the subgenus *Viannia* species and to different degrees in a wide range of other human populations. The number of predicted HLA I-binding epitopes was similar for the different haplotypes [71]. The genetic heterogeneity of human populations is the biggest challenge to the breadth of vaccine coverage [72].

In addition to broad host HLA diversity, the diversity of species causing leishmaniasis can be considered in the development of a leishmaniasis vaccine. It has been shown that pan-specific prototype vaccines with good immunogenicity results in tests with T cells from leishmaniasis patients from different global endemic areas [8]. Therefore, under the pan-specificity criterion, we selected sequences with predicted homology to the maximum number of *Leishmania* species with available sequences.

Knowing that sympatry of different *Leishmania* species is common in endemic regions, the degree of antigen sequence homology among some of the main *Leishmania* species related to human cases in those regions was one of the criteria considered for the identification of potential pan-specific vaccine targets. Interestingly, many of the proteins homologous to the most immunodominant proteins identified in this study were found in subgenus *Viannia* species. This finding corroborates previously reported results that 97.4% of *L. naiffi* genes are orthologous in the subgenus *Viannia* [52]. This work too identified sLnAg, homologous in other species causing CL and VL. We found that the potentially most immunodominant proteins are also the most conserved in *Leishmania*, among the set we analyzed. This result corroborates that homologous epitopes present higher antigenicity, as demonstrated by Gomes-Silva [73].

We propose potential pan-specific anti-leishmaniasis vaccine targets based on abundance, low similarity to human protein sequences, antigenicity, high-affinity binding epitopes for BCR and HLA, and high population coverage, as well as potential conservation by homology in thirteen *Leishmania* species, among the protein sequences in the immunodominant sLnAg fraction. Therefore, conservation and epitope predictions could indicate what experimental effect to expect for sLnAgs sequences composing a human vaccine prototype. An important contribution of this study was the investigation of antigens of the subgenus *Viannia*. We found most of the proteins identified in the databases as “predicted” and “uncharacterized”. In this study, these proteins were identified in the experimental *L. naiffi* sub-proteome.

Among the proteins we have selected from sLnAg, we highlight four groups of homologous proteins in other species that have already been described as vaccine targets or immunogens. (I) The cysteine proteinases have been extensively studied as vaccine targets against experimental cutaneous and visceral leishmaniasis [74,75]. Within this superfamily of proteins, calpain-like cysteine peptidases have been extensively studied in prototype vaccine compositions tested in vivo or in vitro against infection by different *Leishmania* species [76,77,78]. (II) Another potential immunogen already described is composed of circular RNA of a GRIP domain-containing protein, which was able to suppress metastatic breast cancer stem cells in vitro [79]. (III) A SAM protein, on the other hand, whose expression is stimulated by interferon, is also known to be a strong inhibitor of hepatitis B virus replication [80]. (IV) Lastly, a study by Mishra et al. [81] reports that a ribonuclease protein also contributes to the death of *Leishmania* amastigotes in cell culture when enzymatic activity is increased and may be a therapeutic target.

We selected thirteen proteins derived from antigens recognized by antibodies from patients who have developed a curative immune response to cutaneous leishmaniasis, which are potential HLA II and HLA I ligands. However, we found no records in the literature of studies of these proteins in the context of vaccine development other than those we have highlighted.

## 5. Conclusions

This study opens new questions and promising perspectives for the development of vaccines against leishmaniasis, as it is the first to look for vaccine targets in *L. naiffi* species. In conclusion, the *L. naiffi* parasite contains protein antigens that are similar to antigens involved in the resistance response to cutaneous leishmaniasis. According to our in vitro and in silico experiments, these antigens are potential components for a prototype vaccine against leishmaniasis. These molecules may also serve as broad-spectrum diagnostic targets and cure markers. Further studies on each of the selected proteins are required to choose epitopes with greater vaccine and even this potential. The vaccine platform will be described in further work, but our results already enable the application of different platforms, such as subunit vaccines or chimeric poly-epitope, multi-epitope (based on the identified epitomes), or even DNA vaccines.

In the future, we hope a vaccine based on epitopes or subunits of these proteins could serve populations genetically heterogenic from different endemic areas with *Leishmania* species sympatry.

## Figures and Tables

**Table 1:** Immunodominance and antigenicity of the top twenty consensus sequences.

Uniprot Accession	Protein Description	Epitope Prediction (Ligand Epitopes Predicted)						Promiscuity of HLA-Binding Epitope Prediction		Antig *	Length (aa) *
		BCR		HLAII		HLA1		HLAII	HLA1		
		ABC Pred	Bepi-Pred	Consensus	Net-MHCII	Net-CTLpan	Net-MHCpan	Net-MHCII	Net-MHCPan		
**E7BK82**	**A2 protein**	9	1	10	8	2	4	2	2	**0.3**	113
**P0C2T1**	**Ag85A**	35	6	34	109	69	46	23	22	**0.5**	338
**Q7K740**	**CSP**	39	1	28	26	42	30	8	20	**0.9**	397
A0A088RVR2	Uncharacterized protein	155	17	106	483	242	111	26	26	**0.6**	1507
A4HFH6	Putative calpain-like cysteine Peptidase	618	46	361	2406	1172	577	27	26	**0.6**	6164
A0A088RTE8	Calpain-like cysteine peptidase, putative	630	75	501	2248	1376	673	27	26	**0.6**	6169
A0A088RQR0	Uncharacterized protein	310	34	172	1004	436	223	27	26	**0.6**	2972
A0A1E1J8C7	Uncharacterized protein	232	50	226	759	557	337	27	26	**0.6**	2243
A4HFV6	Uncharacterized protein	239	32	135	743	336	223	27	26	**0.6**	2305
A4HBS7	Uncharacterized protein	229	34	332	599	515	390	27	20	**0.5**	2245
A0A088S1Z8	Uncharacterized protein	120	22	95	418	286	190	27	26	**0.5**	1145
A4HFK9	Uncharacterized protein	75	21	122	394	178	110	27	26	**0.6**	757
A4HPC1	Membrane-bound acid phosphatase	53	13	102	391	189	138	25	26	**0.4**	516
A4HF39	Putative mitotubule-associated protein Gb4	264	47	227	954	503	298	27	26	**0.5**	2524
A0A088SJT7	Hsp70 protein-like protein, putative	70	12	154	384	239	145	27	26	**0.4**	724
A4HJ80	Uncharacterized protein	119	14	64	382	179	91	26	26	**0.6**	1150
A4HN04	Uncharacterized protein	108	19	162	355	262	168	25	26	**0.6**	1088
A4HHR1	SAM domain-containing protein	126	32	107	345	196	111	26	26	**0.6**	1238
A4H3R9	Uncharacterized protein	101	8	17	495	25	43	18	20	**0.6**	677
A4HHB1	Uncharacterized protein	99	17	124	431	140	64	27	26	**0.6**	564
A4HAB4	Uncharacterized protein	279	7	208	1024	147	210	27	22	**0.7**	2630
A0A088RMK8	Uncharacterized protein	33	10	90	427	240	160	27	26	**0.7**	1151
A0A1E1J3K6	Uncharacterized protein	93	14	104	388	158	100	27	26	**0.5**	545
A4H7P6	Putative immunodominant antigen	86	21	159	373	170	94	27	26	**0.5**	473
A0A1E1J997	N-acetyltransferase subunit Nat1, putative	70	10	142	284	233	120	27	26	**0.4**	711
A4H7I6	VWFA domain-containing protein	74	15	122	278	246	168	25	26	**0.6**	716
A0A1E1J337	Uncharacterized protein	28	16	44	189	212	130	26	26	**0.4**	757

The identifiers of the control sequences are shown in bold. The sequences of the top twenty proteins with the most epitopes binding to BCRs, HLA I molecules, and HLA II molecules predicted by both programs are represented in dark grey cells. The top twenty sequences with epitopes predicted to bind to two ligand types are shown in medium grey, to one ligand type in light grey. In the last two columns are the antigenicity scores (in bold above the cut-off value) and sequence size values (amino acid). * Abbreviations: Antig means antigenicity; aa means amino acids.

**Table 2:** Summary of potential vaccine targets. Protein sequences selected from the *L. naiffi* sub-proteome by mass spectrometry-based proteomics and sequence mining for pan-specific human leishmaniasis vaccine target candidates. Public databases (Uniprot and Interpro) were used to obtain the main features of the proteins identified in this work as potential vaccine targets.

Uniprot Identifier	Description	Existence	Organism	Family	Length (aa)
A0A088RVR2	GRIP domain-containing protein	Predicted	*L. panamensis*	Golgin	1507
A0A088RTE8	Calpain-like cysteine peptidase, putative	Predicted	*L. panamensis*	Peptidase C2, calpain family (IPR022684); Calpain family cysteine protease (PF00648), and Calpain-like thiol protease family (SM00230)	6169
A0A088RQR0	Uncharacterized protein	Predicted	*L. panamensis*	Myosin heavy chain, non-muscle (PTHR45615)	2972
A4HFV6	Uncharacterized protein	Predicted	*L. braziliensis*	Centrosomal protein 2	2305
A4HBS7	CCR4-NOT transcription complex subunit 1	Predicted	*L. braziliensis*	CCR4-NOT transcription complex subunit 1	2245
A0A088S1Z8	C2H2-type domain-containing protein	Predicted	*L. panamensis*	Not predicted	1145
A4HFK9	Contig, possible fusion of chromosomes 20 and 34	Predicted	*L. braziliensis*	EF-hand domain-containing protein EFHC1/EFHC2/EFHB	757
A4HN04	ATP-dependent RNA helicase	Predicted	*L. braziliensis*	Not predicted	1088
A4HHR1	SAM domain-containing protein	Predicted	*L. braziliensis*	Not predicted	1238
A4HHB1	FH2 domain-containing protein	Predicted	*L. braziliensis*	Not predicted	995
A0A088RMK8	FHA domain-containing protein	Predicted	*L. panamensis*	Not predicted	1151
A0A1E1J3K6	Ribonuclease	Predicted	*L. guyanensis*	RNA-induced silencing complex, nuclease component Tudor-SN	933
A4H7P6	Putative immunodominant antigen	Predicted	*L. braziliensis*	Enhancer of mRNA-decapping protein 4-like	846

## Data Availability

Not applicable.

## Supplementary Materials

The following supporting information can be downloaded at: https://www.mdpi.com/article/10.3390/vaccines11071129/s1, Figure S1: The sLnAg immunoblot membrane with serum from CL cured patients; Table S1: Identified proteins of *L. naiffi* immunodominant fraction; Table S2: Validated immunodominant proteins of *L. naiffi*; Table S3: Similarity between sub-proteome of *L. naiffi* and human proteins.
