# Supplementary material for: Identification of Immunodominant Proteins of the Leishmania (Viannia) naiffi SubProteome as Pan-Specific Vaccine Targets against Leishmaniasis
Source: Vaccines (Basel). 2023 Jun 21;11(7):1129. doi: 10.3390/vaccines11071129 (PMC10386316; doi:10.3390/vaccines11071129)
Supplement: Supplementary file 1 [file vaccines-11-01129-s001.zip › Supplementary Figure S1. Western blot with serum from CL cured patients.pdf]

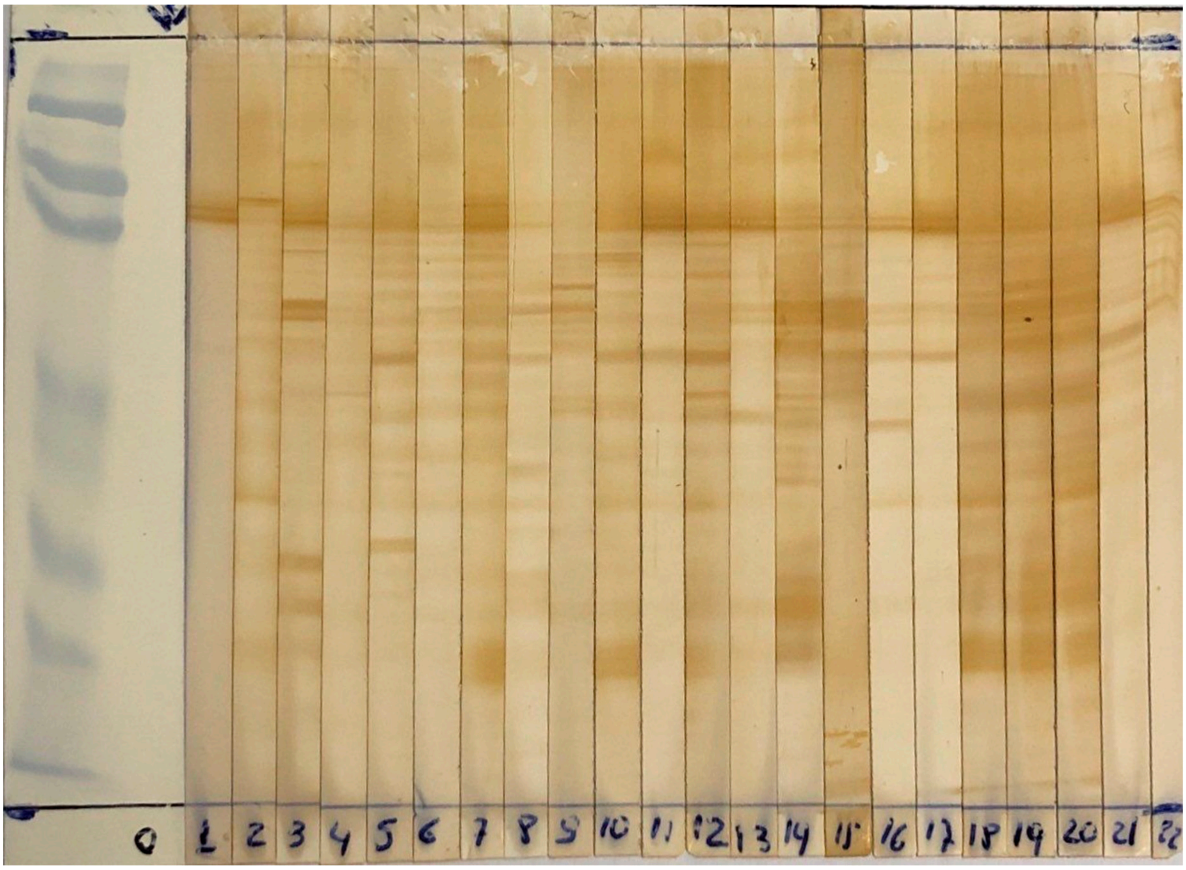

Supplementary Figure S1. The sLnAg immunoblot membrane with serum from CL cured patients. Lane 0: molecular weight range. Lane 1 to 11, 13 and 15 to 18: sLnAg extrat immunobloted with serum from CL cured patients. Lanes 12 and 14: sLnAg extract immunobloted with serum from mucosal leishmaniasis patients (not included in our analysis). Positive control: lanes 19 and 20 (immunoblot incubated with serum samples from active cutaneous leishmaniasis) and negative control: lanes 21 and 22 (incubated with sera samples from patients without leishmaniasis clinic evidence). Lanes 12, 14, 21 and 22 have been removed from the figure 2 because they were from patients with the most severe clinical form of the disease.
